# 

**Published:** 1891-08

**Authors:** 


### CONTENTS FOR AUGUST, 1891.

ORIGINAL COMMUNICATIONS.

Arthrectomy of the Knee-Joint. By HERMAN MYNTER, M. D. .... 1
The Converging Lines of Medical and Surgical Treatment. By NATHAN JACOBSON, M. D... 8
Necrosis of the Ribs, Complicating Pott's Disease. By LOUIS A. WEIGEL, M. D. .... 19

SOCIETY PROCEEDINGS.

New York Academy of Medicine.—Section on Orthopedic Surgery .... 22
Philadelphia County Medical Society .... 30
Buffalo Medical and Surgical Association .... 35
The Medical Society of the County of Erie .... 37

SELECTION.

Aristol .... 50

PROGRESS IN MEDICAL SCIENCE.

Medicine .... 51

EDITORIAL.

Frank Hamilton Potter, M. D. .... 53
Civil Service Examination for Physicians .... 56

PERSONAL.

Dr. A. L. Hummel—Dr. Edwin Walker .... 56
Dr. Jos. Eastman .... 57

SOCIETY MEETINGS.

The American Society of Microscopists—American Neurological Association .... 57
American Dermatological Association .... 58

BOOK REVIEWS.

Materia Medica and Therapeutics .... 59
Text-Book of Hygiene .... 60
International Clinics .... 61
Manual of Hypodermic Medication .... 61
A Treatise on the Diseases of the Nervous System .... 62
Books and Pamphlets Received .... 63

MISCELLANY.

Maltine—An Open Competitive Examination .... 64

